# Collaborative mental health care program versus a general practitioner program and usual care for treatment of patients with mental or neurological disorders in Germany: protocol of a multiperspective evaluation study

**DOI:** 10.1186/s12888-018-1914-5

**Published:** 2018-10-25

**Authors:** Julia Luise Magaard, Sarah Liebherz, Hanne Melchior, Alexander Engels, Hans-Helmut König, Levente Kriston, Holger Schulz, Jeanette Jahed, Anna Levke Brütt, Katrin Christiane Reber, Martin Härter

**Affiliations:** 1 Department of Medical Psychology, Center for Psychosocial Medicine, University Medical Center Hamburg-Eppendorf, Martinistr. 52, Building W26, 20246 Hamburg, Germany; 2 Department of Health Economics and Health Services Research, Center for Psychosocial Medicine, University Medical Center Hamburg-Eppendorf, Hamburg, Germany; 3 AOK Baden-Württemberg, Stuttgart, Germany; 4 Department for Health Services Research, Faculty of Medicine and Health Sciences, Carl von Ossietzky University Oldenburg, Oldenburg, Germany

**Keywords:** Effectiveness, Mental health care, Mental illness, Quality of life, Cost comparison analysis, Integrated care, Collaborative care, Evaluation, Non-randomized controlled trial

## Abstract

**Background:**

German statutory health insurances are pursuing the goal of improving treatment of chronically ill people by promoting networks of health care providers and supporting treatments that reflect the current medical knowledge. The so-called PNP program is a collaborative care program developed by a German statutory health insurance, which defines specific rules on psychiatric, neurological, psychosomatic, and psychotherapeutic treatment. It aims to strengthen provision of guideline-based outpatient treatment and collaboration between different health care providers. It includes the general practitioners’ program, which aims to strengthen the coordinating role of GPs. This study aims to evaluate the PNP program.

**Methods:**

To evaluate the effectiveness of the PNP program, we will conduct a prospective non-randomized controlled trial with primary data comparing patients enrolled in the PNP program and in the general practitioner program (intervention group) to patients enrolled only in the general practitioner program and patients who receive usual care (control groups). To evaluate costs and level of detail of diagnoses in care of patients with PNP program, we will use routinely collected secondary administrative health data in a retrospective quasi-experimental design. Patients who are at least 18 years old, insured by the statutory health insurance AOK, and on sick leave due to one of the mental or neurological diagnoses (affective, anxiety, somatoform or adjustment disorders, alcohol use disorders, schizophrenia, multiple sclerosis) will be included. We will collect data at baseline and at 12-months follow-up. Health-related quality of life (primary data) and direct costs (secondary data) caused by outpatient and inpatient service utilization and medication will be the primary outcomes. We will analyze data using (generalized) linear mixed models and exploratory analyses. We will use entropy balancing to control for possible differences between the groups. We will use an exploratory sequential design including qualitative and descriptive statistical analyses to assess the structure and process quality of the PNP program among health care providers.

**Discussion:**

The results will help to develop a comprehensive picture of collaborative care programs for mental and neurological health care from the perspective of patients, health care providers, and health insurance companies.

**Trial registration:**

German Clinical Trial Register DRKS00013114

**Electronic supplementary material:**

The online version of this article (10.1186/s12888-018-1914-5) contains supplementary material, which is available to authorized users.

## Background

Mental and neurological disorders are common and severe health threats, so they play a decisive role in health care [1, 2]. It is estimated that about 27% of the adult EU population, is or has been affected by at least one mental disorder in the past 12 months [3]. Mental disorders are related to a high degree of personal suffering, disease burden, and health impairment [1, 4]. Several mental disorders, such as depression, alcohol use disorders, bipolar disorders, schizophrenia, and panic disorders, are among the 20 leading causes of disability [2]. Multiple sclerosis, one of the most relevant neurological disorders, is related to impairment of functioning and quality of life [5]. Consequently, mental and neurological disorders represent a major challenge for the health care system and produce high direct as well as indirect costs [6, 7].

The German health care system consists of different health care sectors: primary and specialist care in the outpatient setting, and inpatient care. Statutory or private health insurances cover health care costs; whereby 90% are covered by statutory health insurances. For treatment of mental and neurological disorders, patients can seek GPs, specialist outpatient care from psychiatrists, neurologists, psychotherapists, specialists in psychosomatic medicine as well as inpatient care in neurologic, psychiatric, or psychosomatic hospitals. Although a well-developed mental health care system exists, there is still need for improvement regarding early and correct detection of mental illnesses in primary care and regarding the reduction of intersectoral barriers. For example, GPs can generally identify only about half of the patients with depression and can accurately exclude four of five non-depressed individuals [8]. In addition, there are large delays between detection and adequate treatment of mental disorders [9, 10]. Consequently, patients are on greater risk to maintain high degrees of burden [11–13], lose their motivation to seek treatment or seek treatments that are not sufficiently evidence-based. Even at treatment initiation, speration of the responsibilities of health care providers in differenct sectors impede changing health care sectors (e.g. primary care, specialist care, and inpatient care) [12]. This fragmented mental health care system is a major weakness in Germany [14]. Consequently, overcoming those intersectoral barriers by developing innovative care networks may optimize care of patients with mental and neurological disorders.

To organize patient-centered care, integrated care models may be useful. Clinical practice guidelines recommend stepped and collaborative care models for the treatment of depressive [15] and anxiety disorders [16]. Stepped care aims to tailor evidence-based interventions to patients’ needs, starting with the lowest intensity [17]. In case of non-response, patients receive the next higher level of treatment intensity. Collaborative care aims to strengthen cooperation between different health care providers in order to provide evidence-based and comprehensive treatment.

Studies indicate that stepped and collaborative care models are effective and improve outcomes (e.g. adherence, reduction of depressive symptoms) compared to treatment as usual among patients with depression [18]. A meta-analysis calculated small to medium effect sizes for the effectiveness of collaborative care (depression, mental and physical quality of life, social role function) compared to usual care or with less integrated models (e.g. patient education, consultation-liaison services) among patients with mental disorders [19]. The results of two meta-analyses provide evidence for the effectiveness of collaborative care models compared to care as usual in reducing symptoms among patients with anxiety disorders (standard mean difference = 0.35 95% CI 0.14–0.56) [20] and depression (standard mean difference = 0.34 95% CI 0.25–0.43) [21]. In addition, collaborative care models improve adherence, quality of life, and satisfaction with care among patients with depression [21]. Among patients with multiple scleroses, a long term observational study without control groups did not find effects on disability and health related quality of life [22].

Cost-effectiveness studies showed that the collaborative care models lead either to decreased costs or to slightly increased costs, whereas quality of health care was considerably improved. Although, costs for outpatient care usually increase through collaborative care models, costs for inpatient care decrease [6, 23]. For instance, collaborative care models lead to savings in costs after 2 years among patients with depression [24] and lead to a reduction of acute inpatient treatments among patients with multiple sclerosis [22]. Due to a decrease in days of incapacity to work, indirect costs are reduced among patients with depression [25]. Thus, collaborative care models demonstrate a favorable cost-benefit ratio among patients with mental disorders [26]. However, there is a lack of evidence for the effectiveness of collaborative care programs for patients with multiple sclerosis.

In contrast to other countries, the majority of health care costs in Germany arise from inpatient treatment. To address problems with the fragmented mental health care system in Germany, selective care contracts between particular statutory health insurance companies and health care providers can be concluded in accordance with the German Social Security Code V [27]. These selective care contracts regulate parts of the primary and outpatient care beyond standard care (which is regulated in the collective contract) among people with statutory health insurance. Statutory health insurance companies are committed to provide insurees opportunies for certain selective care contracts (contracts regarding general practitioners-centered care). Health care providers and patients can participate voluntarily in selective care models.

Two selective care programs developed and implemented by the German statutory health insurance AOK Baden Wuerttemberg (AOK BW) are the “general practitioners program” (“HausarztProgramm”) and the “specialist program” (“FacharztProgramm”). Insurees enrolled in the “specialist program” can receive care in the so-called PNP program, if they need care in the fields of psychiatry, neurology, psychosomatics and psychotherapy. The PNP program aims to strengthen collaboration between different health care providers and provision of individualized, guideline-based and biopsychosocial outpatient treatment. Therefore, correct and detailed diagnoses are needed. Differences between components of interventions compared to usual care are described in Table 1. The AOK BW comprehensively implemented the PNP program in Baden-Wuerttemberg (Southwest Germany) and 590,000 patients are receiving care within the specialist program (February 2018). This program needs to be evaluated.

**Table 1:** Differences between components of interventions

	Usual care	General practitioners program	PNP program
Requirements for participation (patients)	- free choice of health care providers with a license in Baden-Wuerttemberg	- minimum time of participation: 12 months - commitment to seek help from GPs enrolled in the program only - commitment to seek help from GPs first (exception: emergencies, gynecologist, ophthalmologists, pediatricians)	- enrolled in the general practitioners program and the specialist program - minimum time of participation: 12 months - commitment to seek help from health care providers enrolled in the program only - commitment to seek help from GPs first (exception: emergencies, gynecologist, ophthalmologists, pediatricians)
Requirements for participation (health care providers)	- license in Baden-Wuerttemberg	- license in Baden-Wuerttemberg - GP	- license in Baden-Wuerttemberg - psychotherapists and specialists in psychiatry, neurology, psychotherapy, or psychosomatic medicine - enrolment in at least one of three modules: psychiatry, neurology, psychotherapy
Role of GPs	- diagnosis, treatment, referral to specialists	- diagnosis, treatment, referral to specialists - guiding through care: structured coordinating and communication to specialists care and merging results of medical examinations	- diagnosis, treatment, referral to specialists - guiding through care: structured coordinating and communication to specialists care and merging results of medical examinations
All providers			
Compensation and diagnoses	- health care services for patients with specific and unspecific diagnoses in accordance with ICD-10 are billable	- health care services for patients with specific and unspecific diagnoses in accordance with ICD-10 are billable - fee for cooperation with specialists	- health care services for patients with predominantly specific diagnoses in accordance with ICD-10 are billable (e.g., for depressive disorders, only the specific codes F32.0-F32.3, F33.0-F33.4 are billable; the unspecific codes F32.8, F32.9, F33.8, and F33.9 are not billable) - fee for cooperation with specialists and GPs
Social service	- social service of the AOK Baden Wuerttemberg	- more structured cooperation between health care provider and social service of the AOK Baden Wuerttemberg	- more structured cooperation between health care provider and social service of the AOK Baden Wuerttemberg
Quality management	- mandatory continuous training courses	- mandatory continuous training courses - participation in quality circles on drug therapy (once per quarter) for GPs	- mandatory continuous training courses - participation in quality circles on drug therapy (once per quarter) for GPs (optional for specialists)
Psychotherapy			
Differences in compensation	- higher payment of regular sessions compared to preparatory sessions (2–4 preparatory sessions)	- higher payment of regular sessions compared to preparatory sessions (2–4 preparatory sessions)	- higher payment for first sessions (acute / initial care = 10/20 sessions) compared to long-term therapy and compared to usual care - higher payment for group psychotherapy compared to usual care - collective incentives for saving sickness benefits
Organization	- review process for approval of long-term psychotherapy	- review process for approval of long-term psychotherapy	- no review process for approval of long-term psychotherapy
Treatment content	- Cognitive Behavioral Therapy - Psychodynamic Psychotherapy - Psychoanalytic therapy - Neuropsychological therapy - Hypnosis - Eye Movement Desensitization and Reprocessing (EMDR)	- Cognitive Behavioral Therapy - Psychodynamic Psychotherapy - Psychoanalytic therapy - Neuropsychological therapy - Hypnosis - Eye Movement Desensitization and Reprocessing (EMDR)	- Cognitive Behavioral Therapy - Psychodynamic Psychotherapy - Psychoanalytic therapy - Additional treatment methods depending on diagnosis: ○ Neuropsychological therapy ○ Hypnosis ○ Eye Movement Desensitization and Reprocessing (EMDR) ○ Systemic psychotherapy ○ Biofeedback ○ Interpersonal therapy
Additional guidelines on accessibility	/	/	- for acute cases: initial session within 3 days; start of psychotherapy within 7 days after established diagnosis - for initial treatment: start of psychotherapy within 4 weeks after established diagnosis
Psychiatry			
Additional compensation	/	/	- additional supplements for → counselling (e.g. supportive counselling for patients with depression) → provision of technical equipment → prescription of discounted medication → collective incentives for the prevention of hospital admissions
Additional guidelines on accessibility	/	/	- limit of waiting time up to 30 min - for acute cases: first doctor’s appointment within the same day
Neurology			
Additional compensation	/	/	- additional supplements for → counselling (e.g. Multiple Sclerosis counselling: 6 units of 20 min per year) → provision of assistants (e.g. with special training on Multiple Sclerosis care)
Additional guidelines on accessibility	/	/	- limit of waiting time up to 30 min - for acute cases: first doctor’s appointment within the same day

In summary, international evidence shows that collaborative care models leads to long-term improvement in patient reported outcomes as well as to reduced costs among patients with mental disorders. To evaluate the benefit of collaborative care models, effects of these models need to be assessed from different perspectives considering various outcome parameters. Nonetheless, multi-perspective and comprehensive evaluations of collaborative care models, like the PNP program implemented in Germany, are currently lacking. Hence, the purpose of this study is to evaluate the PNP program.

## Methods

### Study aims

This study will evaluate the PNP program with regard to (i) effectiveness (patient reported outcomes), (ii) direct and indirect costs, (iii) the level of detail of diagnoses, and (iv) structure and process quality. The study focuses on the health care of patients with the most common mental disorders and the relevant neurological disorder multiple sclerosis.

We will take the perspectives of patients, health care providers, and cost units into account by using patient-related primary and secondary data as well as primary data from health care providers. To evaluate the (i) effectiveness, (ii) costs, and (iii) the level of detail of diagnoses in care with PNP program, we will compare the intervention group, which consists of patients receiving care within the PNP program (intervention group: IG-PNP) with patients, who participate in the “general practitioners program”, but not the PNP program (control group: CG-GP) and with patients, who receive usual care (control group: CG-UC) (Table 1). To evaluate the (iv) structural and process quality of the PNP program, we will survey health care providers enrolled in the PNP program.

### Research questions, hypotheses and study design

#### Effectiveness

*Is treatment within the PNP program more effective compared to the treatment in the control groups (CG-GP, CG-UC) with regard to health related quality of life among patients with mental or neurological disorders (main research question)?*

i.1.) We expect higher scores of health-related quality of life for the IG-PNP compared to the control group CG-GP and to the control group CG-UC.

i.2.) We expect higher scores of functional health, lower illness-specific symptom burden as well as higher patient satisfaction for the IG-PNP compared to CG-GP as well as CG-UC.

*Which groups of patients benefit most from the PNP program?*

i.3.) We expect patients with chronic and severe focus diagnoses to benefit most from the PNP program. Accordingly, we expect chronicity (illness duration) and degree of severity to be significant moderators of the interaction effect. We will investigate additional moderators like gender, age, educational status, diagnoses and comorbidity.

We will conduct a prospective non-randomized controlled trial among patients to answer research questions related to effectiveness. We will include consecutively recruited patients belonging to the three groups (IG-PNP, CG-GP, CG-UC). We will measure health-related quality of life, patient-relevant symptom severity and satisfaction with care at baseline (t_0_, date of sick leave) and at 12-month follow-up (t_1_). Although randomized controlled trials could provide the best evidence in evaluation studies [28], we cannot randomize participants to the three groups for ethical and contractual reasons. In order to prevent the selection bias, we will recruit participants for all three groups in the same area in Germany (Baden Wuerttemberg). We will use entropy balancing to control for potential differences between intervention and control groups. No independent data monitoring committee to determine if the study should be modified or discontinued is necessary, because patients receive interventions irrespective of study continuation or participation. We will monitor and document study responses and discuss possible adaptions if necessary.

#### Direct and indirect costs

*Can participation in the PNP program reduce direct costs for out- and inpatient treatment and costs of medication due to the focus diagnoses?*

ii.a.) We expect costs for out- and inpatient treatment and costs of medication due to the focus diagnoses to be lower among patients in IG-PNP compared to costs for out- and inpatient treatment and costs of medication due to the focus diagnoses among patients in CG-GP as well as CG-UC.

*Can participation in the PNP program reduce indirect costs,* i.e.*, the number of days of incapacity to work due to the focus diagnoses?*

ii.b.) We expect the number of days of incapacity to work and amount of sick pay due to the focus diagnoses to be lower among patients in IG-PNP compared the number of days of incapacity to work and amount of sick pay due to the focus diagnoses among patients in CG-GP as well as CG-UC.

In order to evaluate the direct and indirect costs, we will analyze retrospective secondary data from 2014, 2015, and 2016. Therefore, we will conduct a quasi-experimental study to compare costs caused by service utilization and by sick pay over a period of 12 months between IG-PNP and CG-GP, CG-UC.

#### Level of detail of diagnoses

Is the *amount of specifc diagnoses higher during the treatment process in the PNP program compared to the control group CG-GP and to the control group CG-UC?*

iii.a.) We expect health care providers to submit a higher percentage of specific depression diagnoses (with determination of the degree of severity) in proportion to unspecific depression diagnoses for patients in IG-PNP to the statutory health insurance compared to CG-GP as well as CG-UC.

In order to determine the amount of specific diagnoses, we will also use retrospective secondary data from 2014, 2015 and 2016. Therefore, we will use a trend analysis of diagnoses, stratified according to the intervention and control groups.

#### Structural and process quality

*How do health care providers within the PNP program evaluate the PNP program?*

This is an exploratory evaluation without specific hypotheses.

We will conduct a mixed-methods study with an exploratory sequential design [29] to evaluate the structure and process quality of the PNP program. Therefore, we will include GPs, medical specialists, and psychotherapists working in the PNP program. First, we will conduct semi-structured qualitative interviews with purposefully sampled [30] health care providers working in the PNP program. Second, we will develop an evaluation instrument specific for the PNP program based on the analyzed interviews. Third, all health care providers working in the PNP program will receive this paper-pencil instrument. The present study protocol was prepared in accordance with SPIRIT guidelines [31]. The SPIRIT checklist can be found in Additional file 1.

### Inclusion and exclusion criteria

#### Patients

We will include patients in the study who will be on sick leave due to one of the following eight mental or neurological disorders at the first time during the previous 12 months: bipolar disorders (F31.x), depressive disorders (F32.x, F33.x, F34.1), anxiety disorders (F40.x, F41.x), adjustment disorder (F43.2), somatoform disorders (F45.x), alcohol abuse disorders (F10.x), schizophrenia (F20.x) or multiple sclerosis (G35.x). In addition, patients will be eligible, if they are insured by the AOK BW, are at least 18 years old, and are treated by a health care provider licensed in Baden-Wuerttemberg, Germany. We will exclude patients if they have a legal guardian. Additionally, patients will be excluded if they were on sick leave due to a similar diagnosis to their inclusion diagnosis within the 12 months before the index episode of sick leave. Consequently, mentally ill patients will be excluded if they were on sick leave due to a mental or behavioral disorder (Fxx.x) and patients with multiple sclerosis will be excluded if they were on sick leave due to a G35.x-diagnosis within the last 12 months. Patients choose their contract status (PNP program, “general practitioners program”, usual care) based on their preference.

#### Health care providers

##### Qualitative survey

We will conduct purposeful sampling [30] to cover different perspectives on the PNP program. Therefore, we will include nine interview partners from the three involved health care provider groups (GPs, specialists, psychotherapists). Within the groups of GPs and psychotherapists, we will include interview partners qualified in providing psychodynamic, psychoanalytic, or cognitive behavioral psychotherapy. Within the group of specialists, we will include interview partners enrolled in each of the three modules of the PNP program, namely neurology, psychiatry, and psychotherapy. To focus on potential chances and challenges of combined modules, we will include at least one specialist enrolled in all three modules. Furthermore, we will focus on heterogeneity regarding region, duration of PNP-participation, and gender.

##### Quantitative survey

We will include all health care providers working with adult patients within the PNP program. In February 2018, *N* = 698 health care providers were enrolled in the program.

### Outcome measures

Outcome measures are shown in Table 2.

**Table 2:** Lists of outcome measures

Outcomes	Instruments
*Patient-related primary data*	
*Primary outcomes*	
Health-related quality of life	Short-form health survey (SF-36), mental component summary score [39]
*Secondary outcomes*	
Health-related quality of life	Short-form health survey (SF-36), physical component summary score [39], EuroQol-5D (EQ-5D) [40]
Depressive symptoms	Patient Health Questionnaire – Depression Module (PHQ-9) [41]
Anxiety symptoms	Patient Health Questionnaire – Generalized Anxiety Disorder Module (GAD-7) [42]
Somatoform symptoms	Somatic Symptom Scale – 8 (SSS-8) [43]
Alcohol consumption	Alcohol Use Disorders Identification Test (AUDIT) [44]
Schizophrenic symptoms	Eppendorfer Schizophrenia Inventory – Short Version (ESI-K) [45]
Adjustment disorder-symptoms	Adjustment Disorder-New Module 20 (ADNM-20) [46, 47]
Physical and psychological impact of multiple sclerosis	Multiple Sclerosis Impact Scale (MSIS-29) [48, 49]
Patient satisfaction with outpatient care	Satisfaction with ambulatory care (ZAPA) [50]
*Patient-related secondary data*	
*Primary outcomes*	
Direct costs caused by outpatient and inpatient service utilization and medication within one year following diagnosis	Claims data about outpatient and inpatient service utilization and medication
*Secondary outcomes*	
Indirect costs caused by productivity losses	Days of incapacity to work and sick pay
Level of detail of depression diagnoses	Claims data about diagnoses (specific: F32.0-F32.3, F33.0-F33.4; unspecific: F32.8, F32.9, F33.8, F33.9)
*Health care provider-related primary data*	
Aspects of the PNP program - access - reasons for participation - barriers to participation - advantages and disadvantages of participation - differences in health care - appropriateness of compensation - organizational aspects - interprofessional cooperation - evaluation and use of contract components	Questionnaire on structure and process quality of the PNP program, in-house development based on - qualitative interviews with selected health care providers - a questionnaire from another project (see [51])

### Data collection process

#### Patient-related primary data

The statutory health insurance company (AOK BW) will organize the recruitment in the area of Baden-Wuerttemberg, Germany. First, the statutory health insurance will identify all patients, who meet the inclusion criteria. The statutory health insurance will send information letters, informed consent sheets, and the baseline questionnaires to these patients. Only participants who give their informed consent will be included in the study. Patients, not sending back questionnaires and informed consent sheets, will receive one reminder. The statutory health insurance will transfer group membership and routine diagnoses to the research institution. Twelve month after baseline, participants will receive the follow-up questionnaire. Participants will receive 15 Euro for sending both questionnaires to the UKE.

#### Patient-related secondary data

The statutory health insurance will deliver routinely collected administrative health data retrospectively for 2014, 2015, and 2016 to the research institution.

#### Health care providers-related primary data

To develop a questionnaire for all health care providers within the PNP program, we will interview selected health care providers within the PNP program. Given willingness to participate and obtained informed consent, we will conduct face-to-face-interviews at health care providers’ offices. We will record, transcribe, and anonymize the interviews. We will qualitatively analyze anonymized transcripts in order to prepare a quantitative questionnaire for all health-care providers enrolled in the PNP program. Finally, we will send a study information, informed consent sheets, and this questionnaire to all health care providers within the PNP program. Health care providers will receive financial compensation for participation.

### Sample size calculation

The sample size was calculated for being able to detect a clinically relevant effect regarding the primary outcome health-related quality of life (SF-36) between IG-PNP and control groups CG-GP and CG-UC. Meta-analyses [19, 21, 32] calculated small to moderate effect sizes for collaborative care models. According to Cohen, a small to moderate effect size is defined as d = 0.30 [33]. Given an expected correlation of *r* = 0.4 between outcome at baseline and at follow-up, we will need *n* = 441 patients (*n* = 147 per group) to detect this effect with α = 0.05, power of 0.80 and without the random effect of health care providers offices [34]. Given an expected intraclass correlation of *p* = 0.10 and 100 health care providers in each group, we multiplied the necessary sample size with the design effect of DEF = 1.047 (1 + (147/100–1)*0.10) in consideration of random effects. Consequently, we need to include *n* = 153 patients per group (460 patients in total) in the analyses. We plan to overrecruit by 16.5% to compensate losses of statistical power through possibly different group sizes (IG- PNP ≈ 27%, CG-GP ≈ 22%, CG-UC ≈ 51%) with an allocation ratio of 2.33. Accordingly, we aim to include 536 patients in the analyses. Based on experiences, we expect a response rate of 7% for baseline. We expect dropout rates 40% for follow-up assessments. Consequently, we plan to contact *n* = 12,800 patients to receive data from *n* = 538 patients. We will perform dropout analyses for both study dropout and intervention dropout.

For analyzing direct and indirect costs (ii) and level of detail of diagnoses (iii), we will use complete secondary data from all patients meeting the inclusion criteria in 2015. Because of the exploratory approach, we did not conduct a formal sample size calculation for the evaluation of structure and process quality (iv).

### Statistical analysis

We will use entropy balancing [35, 36] to control for differences between IG-PNP, CG-GP, and CG-UC in primary and secondary data. This method constructs balancing weights for each control observation such that the reweighted control groups match the covariate moments in the intervention group. The weights are based on patient characteristics (e.g. age, gender, region, type of insurance (compulsorily or voluntarily), costs of service utilization, mental comorbidity, and the Combined Comorbidity Index (CCI) [37] from ICD-10 for inpatient stays). The CCI is based on the ICD-10 codes belonging to the hospital stays, in order to take the severity of somatic comorbidities into account.

#### Effectiveness

We will use mixed linear models with fixed effects of group membership (IG-PNP / CG-GP / CG-UC) and further covariates, if necessary (e.g., in case of substantial inbalance between groups) to test the hypotheses regarding effectiveness. Primary medical practice affiliation will be modelled via random effects to adjust for possible clustering. Moderator analyses will be performed by adding interaction terms to the model. Results with *p* < .05 will be considered statistically significant.

#### Cost comparison analysis

We will perform comparisons between IG-PNP and control groups (CG-GP / CG-UC) using generalized linear mixed models. Direct and indirect costs will be considered.

#### Level of detail of diagnoses

To analyze the level of detail of diagnoses, we will conduct exploratory analyses, i.e. descriptive trend analysis, for specific diagnoses in the 12-month follow-up period stratified according to IG-PNP and CG-GP or CG-UC.

#### Structure and process quality

We will evaluate the structural and process quality with a new developed questionnaire and analyze the data descriptively.

### Additional research question

Current developments in scientific discourse on transparency and traceability of study results are likely to require the provision of anonymized primary data sets in the future. The International Committee of Medical Publishers (ICMJE) recommends the responsible provision of primary data from clinical trials [38]. This is to ensure an independent review of scientific results, avoid unwanted repetitions of studies, and thereby meet the moral obligations to patients. In German health care research, this is not the usual practice yet. Usually, researchers provide data in an aggregated form within the framework of scientific publications. To our knowledge the impact of these new requirements and the associated changes to the data protection section on study participants’ willingness to participate have not been systematically investigated yet.

In our evaluation study, we will use the patient-related primary data collection to answer following question:

*Is the participation rate influenced by the level of details in the data that will be made publicly available as described in the study information that participants have to consent to?*

We will compare participation rates of patients receiving one of the three following data protection sections:

1. completely anonymized datasets can be made available in scientific publications
2. completely anonymized datasets can be requested by other scientists for the evaluation of scientific questions; and
3. completely anonymized datasets are exclusively evaluated and stored by the research group.

We expect no differences in participation rates between the three groups.

We will randomly assign all participants to one of the three conditions of data protection sections. We will compare participation rates at the beginning of the study (short-term participation) and after 12 months (long-term participation) between the three conditions.

## Discussion

This multiperspective evaluation study will examine the effectiveness, direct and indirect costs, impact on level of detail of depression diagnoses, as well as structure and process quality of the PNP program among patients with mental and neurological disorders. To the best of our knowledge, this is the first independent and multiperspective study evaluating a comprehensive selective health care program including a collaborative care model for patients with mental and neurological disorders in Germany. This study will counteract the lack of systematic evaluations of complex care models in Germany.

The results are primarily applicable for evaluation of the specific intervention, namely the PNP program. In addition, transferability of the results to other collaborative care models can be examined due to the description of the components of the PNP program. We assume that other collaborative care models include similar components. No conclusions can be drawn about the gain of certain components of the PNP program due to the focus of evaluating a complex intervention. However, the results concerning structure and process quality will generate knowledge about acceptance and feasibility of certain components.

One major strength is the multiperspective approach of this evaluation study. We will evaluate the PNP program taking into account the perspective of patients and health care providers and the health care costs. A collaborative care model adds value to standard care, if it provides improved health related outcomes and satisfaction with care for patients or if it saves costs while patients receive comparable improvements. In addition to the effectiveness and direct and indirect costs, new health care models need to be feasible and acceptable for health care providers. Hence, it is necessary to consider these different perspectives when evaluating those models.

Additionally, we will conduct this evaluation study in routine care, which implies external validity of our results.

Moreover, we will conduct this study with academic and methodological rigor to gain robust results. For instance, we will use instruments with proven psychometric properties. To distinguish between effects on quality of life among all patients and symptom-related outcomes, we will use both generic and illness-specific measurements. Concerning structure and process quality, we will use an elaborate exploratory sequential design [29] combing qualitative and quantitative methods to describe of relevant aspects in a differentiated manner on the one hand and to gain representative results on the other hand.

Instead of randomization, patients choose the intervention based on their preference. This non-randomized design has implications for the internal validity of our study (possible selection and performance bias). To partially control for selection bias, we will perform entropy balancing. Nevertheless, potentially unknown confounders may influence the choice of the program.

In order to investigate the effect of the intervention among patients with a new episode of their disorder, we will include patients who will be on sick leave due to one of the focused mental or neurological disorders at the first time during the previous 12 months. Health insurances receive diagnoses on certificates of incapacity immediately whereas claims data are only available several months after medical appointments. In order to recruit study participants as soon as possible after medical appointments, inclusion relying on diagnoses on certificates of incapacity is the most viable solution. However, participants may have had the same symptoms in previous periods – but without being on sick leave.

Due to limited resources, we will not be able to perform standardized diagnostic interviews to confirm formal diagnoses. Especially in primary care, clinical diagnostics of mental illnesses can differ from standardized diagnostic interviews [8]. We will address this difficulty by including all routine diagnoses from different health care providers and by measuring severity of symptoms with standardized disorder-specific questionnaires.

## Conclusion

Statutory health insurance companies developed and implemented selective care contracts based on collaborative care models in Germany. Nonetheless, systematic evaluations on the effectiveness, cost-effectiveness, quality, and feasibility of these selective care contracts are currently lacking. To develop and evaluate innovative models of health care, the German legislature created the innovation funds of the German Federal Joint Committee. The results of this evaluation study funded by the German Federal Joint Committee will help to develop a comprehensive picture of collaborative care models like the PNP program for mental and neurological health care concerning the perspective of patients, health care providers, and health care costs. In particular, this study will provide suggestions for improvement of the PNP program, transferable to similar collaborative care models and for implementation in usual care. The conclusions drawn from the study are relevant for health care providers and patients, as well as for the legislature and statutory health insurance companies.

## Additional file

Additional file 1: SPIRIT 2013 Checklist. (DOC 121 kb)

## Abbreviations

ADNM-20: Adjustment Disorder--New Module 20
AOK BW: Statutory health insurance AOK Baden Württemberg
AUDIT: Alcohol Use Disorders Identification Test
CCI: Combined Comorbidity Index
CG-GP: Control group with patients receiving care in the “general practitioners program”
CG-UC: Control group with patients receiving usual care
DRKS: German Clinical Trials Register
EQ-5D: EuroQol-5D
ESI-K: Eppendorf Schizophrenia Inventory – short version
GAD-7: Generalized Anxiety Disorder 7
G-BA: German Joint National Committee
GP: General Practitioner
ICD-10: International Classification of Diseases
IG-PNP: Intervention group with patients receiving care within the PNP program
MSIS-29: Multiple Sclerosis Impact Scale
PHQ-9: Patient Health Questionnaire
PNP: Psychiatry, neurology and psychotherapy
SF-36: Short form health survey
SSS-8: Somatic symptom scale – 8
UKE: University Medical Center Hamburg-Eppendorf
ZAPA: Questionnaire of satisfaction with ambulatory care

## References

[1] WHO (2001). The world health report 2001 - mental health: new understanding, new Hope.

[2] World Health Organization (ed.) (2008). The Global Burden of Disease: 2004 Update.

[3] Wittchen HU, Jacobi F (2005). Size and burden of mental disorders in Europe—a critical review and appraisal of 27 studies. Eur Neuropsychopharmacol.

[4] Kessler RC, Aguilar-Gaxiola S, Alonso J, Chatterji S, Lee S, Ormel J, Üstün TB, Wang PS (2009). The global burden of mental disorders: an update from the WHO world mental health (WMH) surveys. Epidemiology and Psychiatric Sciences.

[5] Kobelt G, Lindgren P, Smala A, Bitsch A, Haupt M, Kömel HW, König N, Rieckmann P, Zettl UK (2006). the German cost of MS study group: costs and quality of life of multiple sclerosis in Germany. Eur J Health Econ.

[6] von Korff M, Katon W, Bush T, Lin EH, Simon GE, Saunders K, Ludman E, Walker E, Unutzer J (1998). Treatment costs, cost offset, and cost-effectiveness of collaborative management of depression. Psychosom Med.

[7] Gustavsson A, Svensson M, Jacobi F, Allgulander C, Alonso J, Beghi E, Dodel R, Ekman M, Faravelli C, Fratiglioni L (2011). Cost of disorders of the brain in Europe 2010. Eur Neuropsychopharmacol.

[8] Mitchell AJ, Vaze A, Rao S (2009). Clinical diagnosis of depression in primary care: a meta-analysis. Lancet.

[9] Hermens ML, Muntingh A, Franx G, van Splunteren PT, Nuyen J (2014). Stepped care for depression is easy to recommend, but harder to implement: results of an explorative study within primary care in the Netherlands. BMC Fam Pract.

[10] Katon WJ, Unützer J, Simon G (2004). Treatment of depression in primary care - where we are, where we can go. Med Care.

[11] Shedden-Mora M, Gross B, Lau K, Gumz A, Wegscheider K, Löwe B (2016). Collaborative stepped care for somatoform disorders: a pre–post-intervention study in primary care. J Psychosom Res.

[12] Sachverständigenrat für die Konzertierte Aktion im Gesundheitswesen: Gutachten 2005**:** Koordination und Qualität im Gesundheitswesen. Baden-Baden: Nomos; 2005.

[13] Barkham M, Mullin T, Leach C, Stiles WB, Lucock M (2007). Stability of the CORE-OM and the BDI-I prior to therapy: evidence from routine practice. Psychology and Psychotherapy.

[14] Schulz H, Barghaan D, Harfst T, Koch U (2008). Gesundheitsberichterstattung des Bundes: Psychotherapeutische Versorgung.

[15] NICE (2004). Depression: management of depression in primary and secondary care (clinical guideline 23).

[16] NICE (2004). Anxiety: management of anxiety (panic disorder, with or without agoraphobia, and generalised anxiety disorder) in adults in primary, secondary and community care.

[17] Van Straten A, Tiemens B, Hakkaart L, Nolen WA, Donker MCH (2006). Stepped care vs. matched care for mood and anxiety disorders: a randomized trial in routine practice. Acta Psychiat Scand.

[18] Katon W, Von Korff M, Lin E, Simon G, Walker G, Unützer J, Bush T, Russo J, Ludman E (1999). Stepped collaborative care for primary care patients with persistent symptoms of depression: a randomized trial. Arch Gen Psychiatry.

[19] Woltmann E, Grogan-Kaylor A, Perron B, Georges H, Kilbourne AM, Bauer MS (2012). Comparative effectiveness of collaborative chronic care models for mental health conditions across primary**,** Specialty, and Behavioral Health Care Settings: Systematic Review and Meta-Analysis. Am. J. Psychiatry.

[20] Muntingh ADT, van der Feltz-Cornelis CM, van Marwijk HWJ, Spinhoven P, van Balkom AJLM (2016). collaborative care for anxiety disorders in primary care: a systematic review and meta-analysis. BMC Fam Pract.

[21] Thota AB, Sipe TA, Byard GJ, Zometa CS, Hahn RA, McKnight-Eily LR, Chapman DP, Abraido-Lanza AF, Pearson JL, Anderson CW (2012). Collaborative care to improve the management of depressive disorders: a community guide systematic review and meta-analysis. Am J Prev Med.

[22] Nelles G, Meier U, Limmroth V, Pöhlau D, Wirtz M, Münscher C, Faber B (2010). Integrierte Versorgung Multiple Sklerose – Modellregion Nordrhein. 2-Jahres-Verlaufsbeobachtung. Aktuelle Neurologie.

[23] Simon GE, von Korff M, Ludman E, Katon W, Rutter C, Unützer J, Lin EHB, Bush T, Walker E (2002). Cost-effectiveness of a program to prevent depression relapse in primary care. Med Care.

[24] Katon WJ, Schoenbaum M, Fan MY, Callahan CM, Williams J, Hunkeler E, Harpole L, Zhou XHA, Langston C, Unutzer J (2005). Cost-effectiveness of improving primary care treatment of late-life depression. Arch Gen Psychiatry.

[25] Schoenbaum M, Unützer J, Sherbourne C, Duan N, Rubenstein LV, Miranda J, Meredith LS, Carney MF, Wells K (2001). Cost-effectiveness of practice-initiated quality improvement for DepressionResults of a randomized controlled trial. JAMA.

[26] Van Steenbergen-Weijenburg KM, Van der Feltz-Cornelis CM, Horn EK, Van Marwijk HWJ, Beekman ATF, Rutten FFH, Hakkaart-van Roijen L (2010). Cost-effectiveness of collaborative care for the treatment of major depressive disorder in primary care**.** A sytematic review. BMC.

[27] SGB V: Das Fünfte Buch Sozialgesetzbuch – Gesetzliche Krankenversicherung.; 2017.

[28] Machin D, Campbell MJ (2005). The design of studies for medical research.

[29] Creswell JW (2014). A concise introduction to mixed methods research.

[30] Palinkas LA, Horwitz SM, Green CA, Wisdom JP, Duan N, Hoagwood K (2015). Purposeful sampling for qualitative data collection and analysis in mixed method implementation research. Adm Policy Ment Health Ment Health Serv Res.

[31] Chan A-W, Tetzlaff JM, Altman DG, Laupacis A, Gøtzsche PC, Krleža-Jerić K, Hróbjartsson A, Mann H, Dickersin K, Berlin JA (2013). SPIRIT 2013 statement: defining standard protocol items for clinical trials. Ann Intern Med.

[32] Gilbody S, Bower P, Fletcher J, Richards D, Sutton AJ (2006). Collaborative Care for Depression: a cumulative meta-analysis and review of longer-term outcomes. Ann Intern Med.

[33] Cohen J (1988). Statistical power analysis for the behavioral sciences.

[34] Borm GF, Fransen J, Lemmens WAJG (2007). A simple sample size formula for analysis of covariance in randomized clinical trials. J Clin Epidemiol.

[35] Hainmüller J (2012). Entropy balancing for causal effects: a multivariate reweighting method to produce balanced samples in observational studies. Polit Anal.

[36] Hainmüller J, Xu Y (2013). Ebalance: a Stata package for entropy balancing. J Stat Softw.

[37] Gagne JJ, Glynn RJ, Avorn J, Levin R, Schneeweiss S (2011). A combined comorbidity score predicted mortality in elderly patients better than existing scores. J Clin Epidemiol.

[38] Taichman DB, Sahni P, Pinborg A, Peiperl L, Laine C, James A, Hong S-T, Haileamlak A, Gollogly L, Godlee F: Data sharing statements for clinical trials—A Requirement of the International Committee of Medical Journal Editors In: Mass Medical Soc; 2017. 10.4067/s0034-98872017000600691 29171615

[39] Bullinger M, Kirchberger I (1998). SF-36. Fragebogen zum Gesundheitszustand.

[40] Dolan P (1997). Modeling valuations for EuroQol health states. Med Care.

[41] Spitzer RL, Kroenke K, Williams JBW (1999). The patient health questionnaire primary care study group: validation and utility of a self-report version of PRIME-MD. J Am Med Assoc.

[42] Löwe B, Decker O, Müller S, Brähler E, Schellberg D, Herzog W, Herzberg PY (2008). Validation and standardization of the generalized anxiety disorder screener (GAD-7) in the general population. Med Care.

[43] Gierk B, Kohlmann S, Kroenke K, Spangenberg L, Zenger M, Brähler E, Löwe B (2014). The somatic symptom scale–8 (SSS-8)a brief measure of somatic symptom burden. JAMA Intern Med.

[44] Dybek I, Bischof G, Grothues J, Reinhardt S, Meyer C, Hapke U, John U, Broocks A, Hohagen F, Rumpf H (2006). The reliability and validity of the alcohol use disorders identification test (AUDIT) in a German general practice population sample. J Stud Alcohol.

[45] Maß R (2001). Eppendorfer Schizophrenie-Inventar (ESI). Manual.

[46] Lorenz L (2016). Diagnostik von Anpassungsstörungen. Ein Fragebogen zum neuen ICD-11-Modell.

[47] Einsle F, Köllner V, Dannemann S, Maercker A (2010). Development and validation of a self-report for the assessment of adjustment disorders. Psychology, health & medicine.

[48] Hobart J, Lamping D, Fitzpatrick R, Riazi A, Thompson A (2001). The multiple sclerosis impact scale (MSIS-29). A new patient-based outcome measure. Brain.

[49] Schönberg P (2013). Validierung der deutschen Version der Multiple Sclerosis Impact Scale (MSIS-29).

[50] Scholl I, Hölzel L, Härter M, Dierks M-L, Bitzer EM, Kriston L (2011). Fragebogen zur Zufriedenheit in der ambulanten Versorgung – Schwerpunkt Patientenbeteiligung (ZAPA). Klinische Diagnostik und Evaluation.

[51] Heddaeus D, Steinmann M, Liebherz S, Härter M, Watzke B (2015). Psychenet – Hamburger Netz psychische Gesundheit: Evaluation des Gesundheitsnetzes Depression aus Sicht der teilnehmenden Hausärzte, Psychotherapeuten und Psychiater. Psychiatr Prax.

